# Effectiveness of Eicosapentaenoic and Docosahexaenoic Acid Supplementation for Reducing Uremic Pruritus: A Meta-Analysis of Randomized Controlled Trials

**DOI:** 10.3390/ph19010181

**Published:** 2026-01-20

**Authors:** Chia-An Chou, Lung-Chih Li, Wen-Chin Lee, Chiang-Chi Huang

**Affiliations:** 1 Division of Nephrology, Department of Internal Medicine, Kaohsiung Chang Gung Memorial Hospital, Chang Gung University College of Medicine, Kaohsiung 833, Taiwanherme381981@gmail.com (C.-C.H.); 2 Institute for Translational Research in Biomedicine, Kaohsiung Chang Gung Memorial Hospital, Chang Gung University College of Medicine, Kaohsiung 833, Taiwan

**Keywords:** uremic pruritus, end-stage renal disease, omega-3 polyunsaturated fatty acids, eicosapentaenoic acid, EPA, docosahexaenoic acid, DHA

## Abstract

**Background:** Uremic pruritus is a distressing and common symptom in patients with end-stage renal disease. The development of uremic pruritus involves a multifactorial pathogenesis, including systemic inflammation, dysregulated immune responses, and altered opioid receptor activity. Omega-3 polyunsaturated fatty acids have been reported to mitigate uremic pruritus symptoms. Among omega-3 fatty acids, eicosapentaenoic acid (EPA) and docosahexaenoic acid (DHA) have been reported as potential candidates for alleviating uremic pruritus due to their anti-inflammatory properties. **Methods:** A meta-analysis of seven randomized controlled trials was conducted to evaluate the efficacy of omega-3 supplementation in alleviating uremic pruritus among patients affected with end-stage renal disease. Effect sizes were calculated using Hedges’ g with a random-effects model. Heterogeneity, sensitivity, and meta-regression analyses were performed to explore influencing factors. **Results:** A total of 266 participants were included for analysis. Omega-3 supplementation significantly reduced pruritus severity compared with placebo. Sensitivity analyses were conducted to exclude a single large trial contributing to the results. Meta-regression indicated that higher EPA, DHA, and total omega-3 dosages, and longer treatment duration, were associated with reduced severity of the uremic pruritus than the placebo. No serious adverse events were reported. **Conclusions:** Omega-3 fatty acid supplementation significantly alleviates uremic pruritus in patients with ESRD. These findings support the use of omega-3 fatty acids as a safe and effective adjunct therapy. Further large-scale, long-term trials are warranted to verify these results and assess the long-term effects and safety of omega-3 fatty acids in attenuating uremic pruritus.

## 1. Introduction

Uremic pruritus is a distressing and common symptom affecting patients with end-stage renal disease [1,2,3]. Uremic pruritus significantly diminishes patients’ quality of life and is associated with sleep disruption and emotional distress [4,5]. Its prevalence increases in parallel with declining renal function, from chronic kidney disease to end-stage renal disease (ESRD) [6]. Current evidence suggests that uremic pruritus arises from a multifactorial pathophysiology involving systemic inflammation, xerosis, and dysregulation of the endogenous opioid system [7]. Uremic pruritus typically presents as persistent [6], symmetrical itching [8], frequently involving the back, legs, and arms [9], with minimal primary skin findings apart from excoriations due to scratching [9]. Diagnosis requires the exclusion of other dermatologic or systemic etiologies, along with the evaluation of symptom severity, dialysis adequacy, and metabolic imbalances [10]. Management generally entails a multimodal approach that begins with optimized skin care, particularly routine use of emollients for xerosis, optimization of dialysis, and strict control of calcium-phosphate product or parathyroid abnormalities [10]. In patients with severe and refractory symptoms, systemic treatments such as pregabalin or gabapentin are effective neuromodulators; newer κ-opioid receptor agonists, such as difelikefalin and nalfurafine, have demonstrated substantial therapeutic benefit [9,11,12]. Nevertheless, pruritus may persist or even recur despite treatment. Alternative treatment for uremic pruritus includes gabapentin [13], herbal medicine [14], phototherapy [15], acupuncture [16], and omega-3 fatty acid supplementation [17,18,19,20,21,22,23].

Omega-3 fatty acids, a group of polyunsaturated fatty acids, are considered an essential nutrient [24]. Structurally, the term “omega-3” refers to the position of the first double bond located at the third carbon atom from the methyl (omega) end of the fatty acid chain [25]. The omega-3 fatty acids include alpha-linolenic acid (ALA), docosahexaenoic acid (DHA), and eicosapentaenoic acid (EPA) [24]. ALA is predominantly found in plant-based oils such as chia seed, flaxseed, and canola oil [26], whereas DHA and EPA are abundant in fish oil [27,28]. The terms EPA, DHA, omega-3, omega-3 fatty acid, omega-3 polyunsaturated fatty acid, and fish oil are frequently used interchangeably. Omega-3 fatty acids are commonly consumed as dietary supplements due to their potential benefits for cardiovascular disease prevention [29], triglyceride reduction [24], brain health [29], and anti-inflammation effects [30]. Additionally, omega-3 fatty acids have been investigated as a therapeutic option for uremic pruritus [31].

The definite mechanism by which omega-3 fatty acids attenuate uremic pruritus remains incompletely understood. Several potential pathways have been proposed, including anti-inflammatory effects [30], immunomodulatory [32], peripheral neuropathy [8], and skin barrier integrity [33]. Among these, systemic inflammation is considered a key contributor to the development of uremic pruritus. Supporting this notion, patients with ESRD frequently exhibit elevated serum levels of pro-inflammatory cytokines and interleukins (IL), including IL-2, IL-6, IL-31, C-reactive protein, and tumor necrosis factor alpha (TNF-α) [10]. Omega-3 fatty acids counteract inflammation by serving as precursors to anti-inflammatory lipid mediators [31]. These mediators include resolvins, protectins, and maresins [34], which inhibit neutrophil activity and cytokine release [35]. EPA and DHA also compete with arachidonic acid for cyclooxygenase and lipoxygenase enzymes [36], thereby reducing the production of pro-inflammatory prostaglandins and leukotrienes [37]. These anti-inflammatory eicosanoids help mitigate both systemic and cutaneous inflammation, driving itch in uremic pruritus. Clinical studies have shown that supplementation with omega-3 fatty acids can decrease pruritus intensity and inflammatory markers such as CRP and IL-6, though some trials reported no significant improvement possibly due to small sample sizes and heterogeneous designs [22,38]. Moreover, omega-3 fatty acids can integrate into epidermal lipids to improve skin hydration and barrier integrity, thereby reducing xerosis, a common contributor to pruritus in dialysis patients [39,40]. Overall, omega-3 fatty acids appear to alleviate uremic pruritus through integrated anti-inflammatory, immunomodulatory, and skin-protective mechanisms.

Meta-analyses about the role of the omega-3 fatty acids have been reported [31,41,42]. These analyses report that omega-3 fatty acids are associated with a decreased severity of uremic pruritus. Nevertheless, specific omega-3 fatty acids, such as EPA and DHA, and their effects on attenuating uremic pruritus are not fully explored by these meta-analyses. None of these meta-analyses include trials after 2022. To understand the role of DHA and EPA in the decreased incidence of uremic pruritus. A meta-analysis was conducted as the primary aim of this work. Updated clinical trials, which were published after the release of the previous meta-analyses [31,41,42], were also included to increase the robustness of this work.

## 2. Materials and Methods

### 2.1. General Methods of Meta-Analysis

The meta-analysis was conducted subject to the PRISMA 2020 guidelines [43]. This meta-analysis and its protocol were registered in International Database to Register Evidence Synthesis Projects (registration number: INPLASY2025110017). Ethics review board approval or participant informed consents were not required for this analysis.

### 2.2. Article Search, Identification, and Filtering

The literatures were searched from EMBASE, PubMed, Cochrane Central, and Clinicaltrials.gov. The Boolean logic keywords ([Fish Oils] OR [Omega-3 Fatty Acids] OR [n-3 fatty acid] OR [Eicosapentaenoic Acid] OR [EPA] OR [Docosahexaenoic Acids] OR [DHA] AND [Pruritus] OR [itch] OR [uremic pruritus] OR [Chronic Kidney Failure] OR [Renal Insufficiency] OR [end-stage renal disease] OR [ESRD] OR [uremia] OR [uraemia]) were used. The field tags for searching the medical subject heading (MeSH), synonyms, and limiting search words found only in the title or abstract were used for all databases. The time span of all literature searched from databases was between January 1965 and October 2025. The full search strategies and corresponding keywords for each database are provided in Supplementary Table S2. Two authors (Chou, C.-A., and Huang, C.-C.) performed the search and identified relevant articles for this meta-analysis independently. References of review articles relevant to this meta-analysis were also reviewed [31,41,42]. For studies on which the two authors were unable to reach consensus regarding inclusion, the third study author, Li, L.-C., was consulted for the verdict. No language restrictions were imposed during the search.

### 2.3. Inclusion and Exclusion Criteria

The PICO (population, intervention, comparison, outcome) setting was used for this meta-analysis. Human participants, omega-3 fatty acid supplement, placebo, and changes in pruritus scores were set for P, I, C, and O, respectively. The inclusion criteria of this meta-analysis were: (1) randomized controlled trials, (2) studies including human participants with ESRD, (3) studies containing at least one quantitative outcome to measure the severity of uremic pruritus before and after omega-3 fatty acid supplement, and (4) studies containing a placebo group for comparison. The exclusion criteria of this meta-analysis were: (1) non-randomized-controlled trials, (2) studies lacking quantitative measurements, (3) studies not containing a placebo-controlled group, and (4) studies including participants overlapped with other published trials for the same topic.

### 2.4. Methodological Quality Assessment

The methodological quality appraisal of the studies for this meta-analysis was conducted according to the Cochrane Risk of Bias tool version 2 (RoB 2) [44]. Bias domains, including bias of the randomization process, deviations from intended interventions, missing outcome data, measurement of the outcome, selection of the reported result, and overall bias were assessed as the Rob 2 described [44]. In this meta-analysis, a per-protocol analysis was selected for meta-analysis from the intervention adherence section of RoB 2.

### 2.5. Primary Outcome

The primary outcome was the change in pruritus scores by omega-3 fatty acid supplement and placebo. For studies reporting multiple scores for evaluating the pruritus severity, the score was selected based on the measure deemed most representative within the context of each study, as determined by consensus between two authors (Chou, C.-A. and Huang, C.-C.). If consensus could not be reached, a third author (Li, L.-C.) was consulted to provide the final decision.

### 2.6. Secondary Outcome

The secondary outcome of this meta-analysis was the rate of adverse events. For studies with zero adverse events, the value 0.5 was applied instead of 0 for calculation in the software Comprehensive Meta-Analysis [45].

### 2.7. Data Extraction and Converting

Data for this meta-analysis, including basic demographic data, treatment duration, pruritus scores, doses of omega-3 fatty acids and placebos, and adverse events, were transcribed from the original studies [17,18,19,20,21,22,23]. Data extracting, converting, merging means, and merging standard deviations from the original studies [17,18,19,20,21,22,23] were processed according to the recommendations of the Cochrane Handbook for Systematic Reviews of Interventions [46,47,48]. When multiple post-treatment time points were reported, the longest duration of follow-up was selected for analysis. For crossover studies [17,21], only data from the first treatment period, prior to crossover, were included in the analysis.

### 2.8. Statistical Analyses and Software

The meta-analysis was conducted, and figures were exported using Comprehensive Meta-Analysis software (version 3, Biostat, Englewood, NJ, USA) [49]. A random-effects model was applied, and statistical significance was defined as a two-tailed *p*-value < 0.05. Hedges’ *g* with corresponding 95% confidence intervals (CIs) was used to present the primary outcomes in the meta-analysis. For the secondary outcomes, odds ratios along with 95% CIs were calculated and illustrated. *I*^2^ statistics were used to examine the degree of heterogeneity of the included studies. *I*^2^ with a value of 25, 50, and 75% was defined as low, moderate, and high heterogeneity, respectively. Subgroup analysis, meta-regression, funnel plots and sensitivity analysis using the one-study removal method were processed and plotted using Comprehensive Meta-Analysis software. For the meta-regression analyses, the doses of EPA, DHA, the combined EPA+DHA dose, and duration of treatment were entered as moderator variables in Comprehensive Meta-Analysis software. Meta-regression was then performed to evaluate the relationship between each moderator and its effect size.

## 3. Results

### 3.1. Study Identification

**Figure 1:**
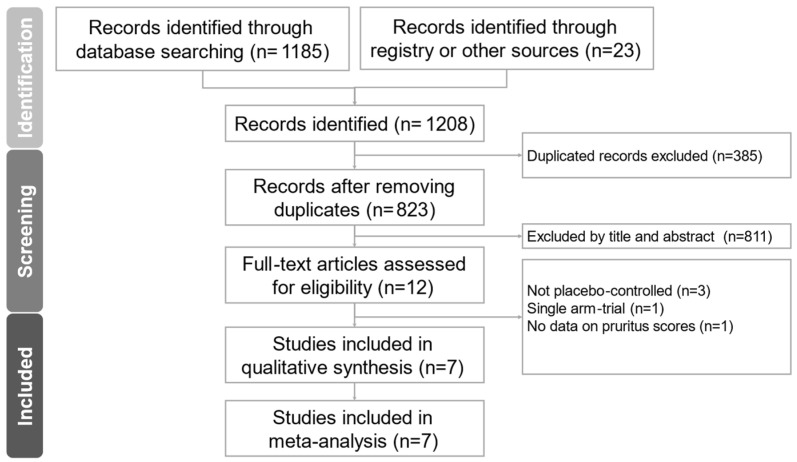
PRISMA 2020 flowchart for meta-analysis. The figure depicts the flowchart for literature filtering in this meta-analysis. A total of 1208 articles were identified from PubMed, EMBASE, Cochrane Central, and Clinicaltrials.gov. After filtering duplicate records and removing articles that did not meet the inclusion criteria, a total of seven articles are eligible for analysis.

Using the PRISMA (Preferred Reporting Items for Systematic reviews and Meta-Analyses) literature review [43], seven articles were identified and enrolled for meta-analysis [17,18,19,20,21,22,23]. The study identification pipeline is shown in Figure 1. From PubMed, EMBASE, Cochrane Central, and Clinicaltrials.gov, 1208 articles were identified. After removing duplicate studies and articles by reviewing titles and abstracts, twelve articles were identified. Among them, five articles were excluded because trials lacked a placebo, a control group, or continuous variables in results for analysis [36,50,51,52,53]. The details of exclusion of these five articles are described in Table S1 [36,50,51,52,53]. Details of data extraction from the seven randomized control trials are listed in Table 1. These seven randomized controlled trials enrolled for analysis include a total of 266 participants with a mean age of 58.19 ± 12.67 (standard deviation) years. The study duration ranged from twenty days [22] to sixteen weeks [20]. Study diagnoses in these seven trials were all end-stage renal disease, and subjects in six trials were treated with hemodialysis [17,18,19,20,22,23], and one with peritoneal dialysis [21] (Table 2). The time points and periods used for the meta-analysis are listed in Table 2. These seven trials revealed moderate heterogeneity (*I*^2^ = 49.99%).

### 3.2. Methodological Quality of the Included Studies

**Figure 2:**
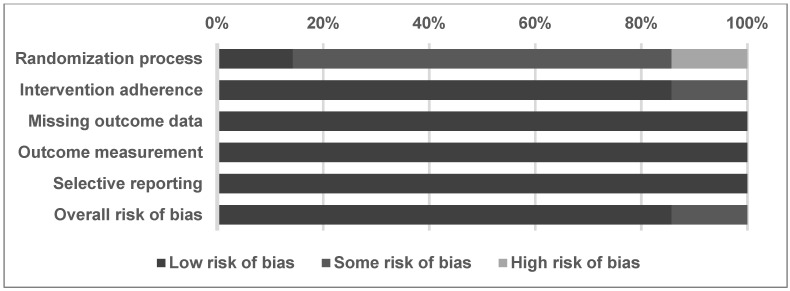
Summary of quality assessment of studies included in the meta-analysis using Cochrane Risk of Bias tool for randomized trials (RoB 2). The figure shows the assessment of risk of bias in the enrolled trials and the distribution of the risk level. Six items of the risk of bias are shown in the figure. The percentages of low, some, and high risk of bias for each item are shown in the figure.

The overall methodological quality of the included studies is evaluated using the Cochrane Risk of Bias tool for randomized trials (RoB 2), as described in Table 3 [44]. Most of the included trials raised concerns due to insufficient details about randomization, which may affect the certainty of the pooled analysis. In total, 85.7% of the evaluated studies had some risk of bias, and 14.3% had low risk of bias in the overall risk of bias domain (Figure 2). In a detailed assessment, one study was rated as having a low risk of bias for randomization [23], five as having some risk due to insufficient details of concealment [17,18,19,20,22], and one as having a high risk because of imbalanced control and intervention groups [21]. One study was rated as having some risk of intervention adherence due to insufficient details on double-blind interventions, which raises concern about participants’ awareness of the interventions [18]. The remaining six trials were rated as having a low risk of adherence to the intervention. Regarding the risk of bias of missing outcome data, outcome measurement, and selective reporting, these seven trials were rated as having low risk [17,18,19,20,21,22,23]. The risks of bias assessment are detailed in Table 3.

### 3.3. Primary Outcome: Effects of Omega-3 Fatty Acid on Uremic Pruritus

**Figure 3:**
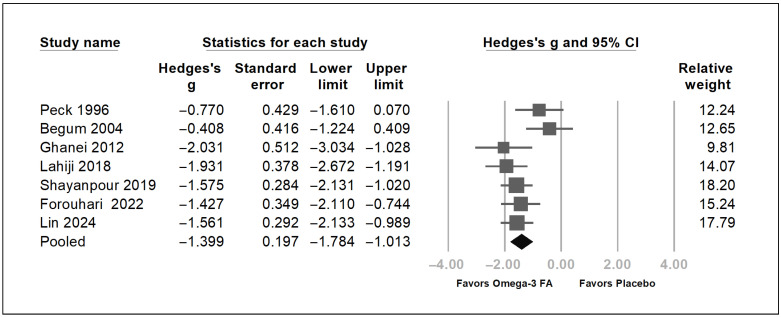
Forest plot of the effects of omega-3 fatty acids on uremic pruritus as compared with the placebo. The grey square indicates individual study results, with size indicating weight. The grey horizontal line indicates 95% confidence intervals. The black rhombus indicates the overall pooled effect. The forest plot of the effects of omega-3 fatty acids on pruritus score as compared with placebo using random-effects models. The sample sizes of enrolled trials are 25, 22, 22, 40, 64, 33, and 60 by Peck 1996 [19], Begum 2004 [20], Ghanei 2012 [22], Lahiji 2018 [21], Shayanpour 2019 [23], Forouhari 2002 [17], and Lin 2024 [18], respectively. The Omega-3 fatty acids showed a significant reduction in pruritus score. CI, confidence interval. FA: fatty acid.

**Figure 4:**
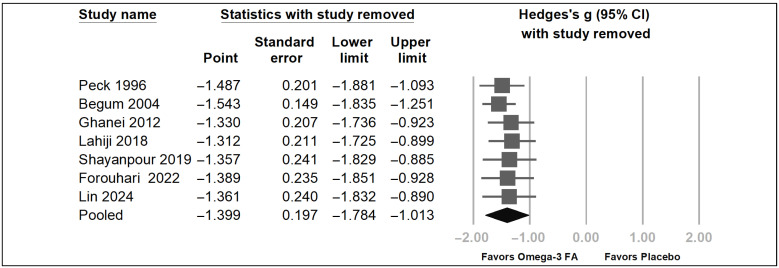
Sensitivity analysis using the one-study removal of omega-3 fatty acids on uremic pruritus as compared with the placebo [17,18,19,20,21,22,23]. The results of a sensitivity analysis using the one-study removal method using random-effects models. The result did not change significantly by removing any one of the included trials. All analyses show a statistical reduction in pruritus score by omega-3 fatty acids as compared with the placebo. CI, confidence interval. FA: fatty acid. The grey square indicates individual study results, with size indicating weight. The grey horizontal line indicates 95% confidence intervals. The black rhombus indicates the overall pooled effect.

**Figure 5:**
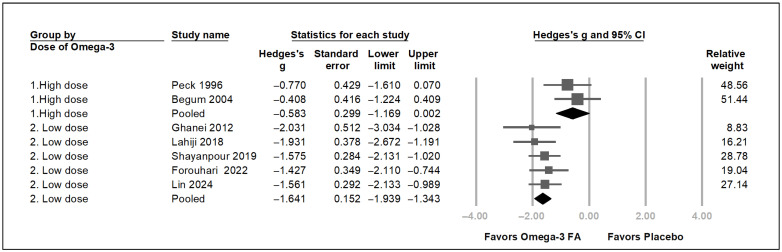
Subgroup analysis grouped by dose of omega-3 fatty acid. The forest plot of subgroup analysis using the dose of omega-3 free fatty acids using the random-effect models. Studies using doses of the omega-3 fatty acid over 3 g per day are considered high-dose studies, and vice versa [17,18,19,20,21,22,23]. The directions of association between the use of a dose of omega-3 fatty acid and pruritus score are greater in the low-dose study group than in the higher-dose group. CI, confidence interval. FA: fatty acid. The grey square indicates individual study results, with size indicating weight. The grey horizontal line indicates 95% confidence intervals. The black rhombus indicates the overall pooled effect.

Overall, these seven trials demonstrated a statistically significant reduction in uremic pruritus score [17,18,19,20,21,22,23] (Hedges’ *g* = −1.399, 95% CI = −1.784 to −1.013, *p* < 0.001) (Figure 3). A moderate heterogeneity was found (*I*^2^ = 49.99%). Sensitivity analysis was performed using the one-study removal method to assess the influence of individual studies on the pooled estimate (Figure 4) [54]. The omega-3 fatty acids showed an absolute reduction in the 5D pruritus score of 1.8 by Shayanpour et al. [23] and 9.2 by Lin et al. [18], an absolute reduction in the visual analog pruritus scale of 3.4 by Forouhari et al. [17], an absolute reduction in the uremic pruritus score of 1.6 by Peck et al. [19], 7.8 by Begum et al. [20], 13.9 by Ghanei et al. [22], and 3.1 by Lahiji et al. [21]. After removing each of these seven trials, the overall trend of the Hedges’ *g* still demonstrated a statistically significant reduction in uremic pruritus score (Figure 4). This one-study removal test showed that the pooled results were not caused by a single study with a considerably high weight. To identify factors associated with omega-3 fatty acid effects on uremic pruritus, subgroup analysis was performed. Because the dose of omega-3 fatty acid of 4 g per day or more is considered a therapeutic dose [56], a daily dose of omega-3 fatty acids of 4 g per day or more was defined as the high-dose group, whereas a dose of less than 4 g per day was defined as the low-dose group. As depicted in Figure 5, the low-dose omega-3 fatty acid group showed a significant decrease in uremic pruritus score; in contrast, the high-dose omega-3 fatty acid group showed only a borderline decrease in uremic pruritus score. The high-dose omega-3 fatty acid did not show a more apparent effect on decreasing uremic pruritus than the low-dose one. Within the limited dose range and small number of studies, a clear dose–response pattern at the level of total omega-3 dose could not be demonstrated.

**Figure 6:**
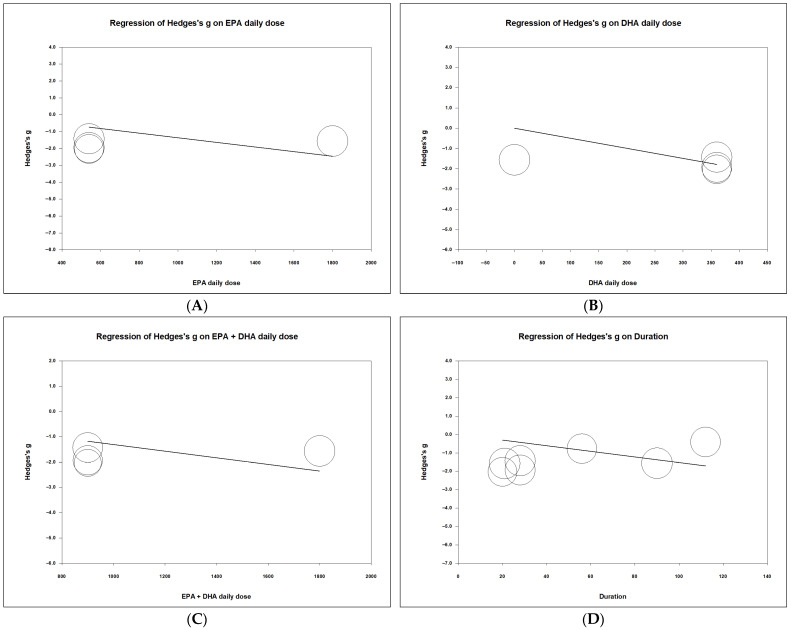
Meta-regression of Hedges’ g on daily dose of eicosapentaenoic acid, docosahexaenoic acid, sum of eicosapentaenoic acid and docosahexaenoic acid, and treatment duration. The figures depict the meta-regression of Hedges’ g on (**A**) daily EPA dose, (**B**) daily DHA dose, (**C**) daily EPA + DHA dose, and (**D**) treatment duration. On the horizontal axis, EPA and DHA are expressed as daily doses (grams per day); duration is expressed in days. EPA: eicosapentaenoic acid. DHA: docosahexaenoic acid.

As described in Figure 3 and Figure 5, the omega-3 fatty acid is associated with decreased severity of pruritus with unclear correlation of its dose. It is hypothesized that the specific omega-3 fatty acids, EPA and DHA, are the effective components to attenuate the uremic pruritus. To test this hypothesis, exploratory analyses were conducted to determine whether EPA or DHA is the effective component in omega-3 fatty acids for alleviating uremic pruritus. Meta-regression analyses were performed. Four studies documenting the dose of DHA and EPA were included for meta-regression [17,18,21,22]. As depicted in Figure 6A,B,C, the dose of EPA, DHA, and the sum of EPA and DHA were negatively correlated with the severity of the uremic pruritus (coefficient β = -0.0014 per mg, *p* = 0.0184; coefficient β = −0.005 per mg, *p* = 0.0066, coefficient β = −0.0013 per mg, *p* = 0.0001, respectively). In exploratory analyses, higher EPA and DHA doses appeared to be associated with greater reductions in pruritus, but these findings are based on a few trials and should be interpreted with caution.

To understand the association between the treatment duration of the omega-3 fatty acid and the severity of uremic pruritus, an exploratory post hoc meta-regression analysis was performed. The duration of the omega-3 fatty acid treatment was negatively correlated with the uremic pruritus scale (coefficient β = −0.0156 per day, *p* = 0.0379) (Figure 6D). The result suggests that omega-3 fatty acid treatment duration is associated with pruritus scores.

### 3.4. Secondary Outcome: Adverse Events of Omega-3 Fatty Acids

In these seven enrolled trials, a total of 128 participants received omega-3 fatty acids. No trial reported treatment-related adverse events; however, several studies did not formally assess adverse events [17,18,19,20,21,22,23]. Treatment-associated adverse event rates (Supplementary Figure S1) did not show a statistically significant difference between groups (odds ratio (OR) = 1.88, 95% CI = 0.24 to 4.90, *p* = 0.913).

## 4. Discussion

This meta-analysis demonstrates that omega-3 fatty acids reduce the severity of uremic pruritus in patients with ESRD. This result is consistent with previous reports [41,42]. In this work, the latest trial was included to increase the number of trials [18], thereby enhancing the statistical power of the meta-analysis. Also, meta-regression was used in this meta-analysis to identify the confounding factors that affect the uremic pruritus.

In this meta-analysis, seven trials were included. Previous meta-analyses used the standardized mean difference as the effect size indicator [41,42], whereas in this work, Hedges’ g was used. The reason for using Hedges’ g in this work is that Hedges’ g is used to express the difference between two group means in standard deviation units and was specifically developed to correct the bias resulting from a small sample size, particularly when the sample size is less than 10 [57]. As described in Table 2, the sample size of the work by Peck et al. is less than 10 [19], which justifies using Hedges’ g for the main analysis. This meta-analysis showed moderate heterogeneity (*I*^2^), which presumably results from small sample sizes and consequent increased standard deviations, as well as different quantification methods for outcome measurement.

Uremic pruritus is common in patients with advanced chronic kidney disease. It significantly impacts quality of life, including sleep disturbance, depression, and skin damage from scratching. The pathophysiology is multifactorial and not fully clarified. Nevertheless, it is still proposed that possible mechanisms include systemic inflammation and dysregulated cytokines, elevated levels of pruritogens, including histamine and opioids, and an imbalance in essential fatty acids and lipid mediators. According to the first randomized report that omega-3 fatty acids alleviated the uremic pruritus, it was proposed that the deficiency of essential free fatty acids leads to dry and scaly skin [19]. In the same study, patients with ESRD had lower baseline concentrations of eicosatrienoic acid (20:3n-9), arachidonic acid (20:4n-6), and eicosapentaenoic acid (EPA, 20:5n-3), and higher oleic acid (18:1n-9) concentration than normal subjects [19]. EPA, a precursor of prostaglandin E2 (PGE2), is regarded as an anti-inflammatory prostaglandin [18,19,20,21]. Peck et al. argued that supplementation with omega-3 fatty acids enriched in EPA attenuated inflammation through these metabolic pathways, thereby alleviating uremic pruritus [19]. However, in another study by Begum et al., the EPA (20: 5n-3) only showed a marginal increase without statistical significance by omega-3 fatty acid supplement, but docosapentaenoic acid (DPA, 22:5n-3) and docosahexaenoic acid (DHA, 22:6n-3) were increased by omega-3 fatty acid supplement [20]. Because DPA, DHA, and EPA all belong to the omega-3 fatty acid [24], the divergent results of the fatty acid profiles between Begum et al. [20] and Peck et al. [19] might be attributed to the different components of the omega-3 fatty acids. Nevertheless, these results are still consistent with this meta-analysis that omega-3 fatty acid was associated with lower pruritus scores. Comparing the effect of uremic pruritus between omega-3 fatty acids and gabapentin, treating ESRD patients with omega-3 fatty acids or gabapentin resulted in the same median reduction of 5D scale from 13 to 7 [49]. This surrogate evidence parallels our finding that omega-3 fatty acids were associated with lower pruritus scores.

The subgroup analysis showed that the high-dose omega-3 fatty acid was not associated with a more significant effect on attenuating uremic pruritus than the low-dose. This result might be attributed to the heterogeneity of the enrolled trials. Differences in dialysis vintage, comorbidities, dosages of omega-3 fatty acids, and components of omega-3 fatty acids among the enrolled trials may contribute to the paradoxical results. As mentioned above, specific omega-3 fatty acids, such as DPA, EPA, and DHA, were increased by omega-3 fatty acids. This result also raised the conjecture that only particular omega-3 fatty acids or their specific metabolite are effective in alleviating uremic pruritus.

Among omega-3 fatty acids, DHA and EPA have been studied for their anti-inflammatory effects. These two omega-3 fatty acids were negatively correlated with pruritus severity. Although the mechanism by which omega-3 fatty acids attenuate uremic pruritus is not fully understood, according to Peck et al., in patients with ESRD treated with omega-3 fatty acids, the EPA (20:5n-3) concentration significantly increased compared with those treated with a placebo [19]. Peck et al. argued that ESRD patients with low arachidonic acid concentrations were indicative of abnormal eicosanoid formation and proposed that omega-3 fatty acids increased the anti-inflammatory PGE3 [19]. Alternatively, in an animal study, the pro-inflammatory prostaglandins and their metabolites—prostaglandin E2 (PGE2), prostaglandin F2α (PGF2α), prostaglandin D2 (PGD2), thromboxane B2 (TXB2), 5-hydroxyeicosatetraenoic acid (5-HETE), and arachidonic acid [55,56,57,58,59,60,61,62,63,64,65]—and the anti-inflammatory ones—15-hydroxyeicosapentaenoic acid (15-HEPE), 18-hydroxyeicosapentaenoic acid (18-HEPE), and resolvin E1 (RvE1) [66,67,68]—were both increased in the skin tissue [69]. Of note, in this study, the anti-inflammatory 15-HEPE and 18-HEPE in skin tissue were drastically increased in mice fed EPA compared with the control group [68]. This result is consistent with that of 15-HEPE and 18-HEPE, both metabolites of EPA [70]. Therefore, the anti-pruritus effect of EPA might be mediated by its metabolites. Clinically, Lin et al. reported that supplying ESRD patients with omega-3 fatty acids containing EPA with a purity of over 90% significantly decreased pruritus scores [18,36]. These results also support that EPA is a specific omega-3 fatty acid that alleviates uremic pruritus. Similarly, DHA, a metabolite of EPA [30], also exhibits anti-inflammatory effects. In an atopic dermatitis murine model, administering DHA suppressed activation or proliferation of helper T cells and lowered the serum IgE level [71]. Collectively, EPA and DHA might attenuate skin pruritus through immune remodeling.

Regarding the adverse effects, overall, omega-3 fatty acids are safe and well-tolerated [72]. A meta-analysis showed no statistical difference in the adverse event of skin disorder between prescription omega-3 fatty acid and generic omega-3 fatty acid [73]. However, in another meta-analysis including 21 randomized trials, prescription omega-3 fatty acid products were associated with skin abnormalities, including eruption, itching, exanthema, or eczema [72]. Nevertheless, in the same analysis, subgroup analysis showed that the EPA/DHA combination products were only associated with increased risk of gastrointestinal adverse events [72]. Furthermore, some evidence suggests the EPA-only regimen is not associated with an increased risk of gastrointestinal and skin disorders [72]. This result is also plausible, as not all omega-3 fatty acids are necessary for alleviating skin itching. This result was also indicative that each omega-3 fatty acid might have a different, or even opposite, effect on the purpose, such as skin pruritus. While no major safety signal emerged, the current evidence base is too small to exclude rare or delayed adverse effects.

Several limitations remain in this study. First, the pruritus scores used across trials varied, which might cause heterogeneity, although Hedges’ g was introduced to measure the effect size across trials with different score systems. Second, to measure the effects of DHA and EPA, only four trials reported the doses of EPA and DHA, and this small sample size can have low statistical power and wide uncertainty. Third, the overall duration of the omega-3 fatty acid intervention period is relatively short. Only two trials implemented the omega-3 fatty acid intervention for three months or longer. As uremic pruritus usually presents as a chronic symptom [6], this relatively short omega-3 fatty acid intervention period may be insufficient to demonstrate its long-term effects, which warrant further investigation. Fourth, because unavailable information about intention-to-treat, per-protocol analysis was chosen, this analysis method may overestimate the benefit of omega-3 fatty acid supplementation. Fifth, the use of multiple pruritus scales across trials, including visual analog scale, 5D pruritus score, and Duo score, can introduce substantial measurement bias. Finally, the types and severity of the adverse effects were not specified in these trials. The relatively low participant number may have underpowered and understated the impact of the adverse effect.

In conclusion, omega-3 fatty acids demonstrated a statistically significant effect on pruritus compared with placebo. The effect was statistically significantly correlated with treatment duration, EPA dose, and DHA dose, but evidence is limited by small sample reporting. Future studies are needed to assess the long-term benefits and safety of omega-3 fatty acids for the treatment of pruritus in ESRD patients.

## Figures and Tables

**Table 1:** Summary of the retrieved trials investigating the effect of omega-3 fatty acids on alleviating pruritus in the enrolled participants. The table describes the seven trials included in the meta-analysis. Key information on the study designs is summarized in the table.

First Author and Year	Country	Population	Participants (Female/Male)	Age ^1^	Study Design	Allocation Concealment	Randomization
Peck 1996 [19]	USA	ESRD under HD	Omega-3: 3/5 ^2^ Olive oil: 5/4 ^2,4^ Safflower oil: 4/4 ^2,4^	54.8 ± 16.2 45.6 ± 17.4 49.5 ± 17.2	RCT, double-blind	Not mentioned	Not mentioned
Begum 2004 [20]	USA	ESRD under HD	Omega-3: 3/7 ^2,5^ Placebo: 6/6 ^2,5^	61.2 ± 19.42 49.25 ± 18.12	RCT, double-blind	Not mentioned	Not mentioned
Ghanei 2012 [22]	Iran	ESRD under HD	Omega-3: 3/8 ^2^ Placebo: 5/6 ^2^	59.90 ± 14.82 53.09 ± 13.08	RCT, double-blind	Not mentioned	Not mentioned
Lahiji 2018 [21]	Iran	ESRD under CAPD	Omega-3: 11/9 ^2^ Placebo: 10/10 ^2^	62.1 ± 11.6 61.9 ± 10.8	RCT, double-blind	Not mentioned	Not mentioned
Shayanpour 2019 [23]	Iran	ESRD under HD	Omega-3: 5/27 ^2^ Placebo: 9/23 ^2^	51.91 ± 6.58 56.25 ± 8.86	RCT, double-blind	Blocks of six for allocation concealment	Blocks of six for allocation concealment
Forouhari 2022 [17]	Iran	ESRD under HD	Omega-3: 5/12 ^3^ Placebo: 5/11 ^3^	59.00 ± 13.56 ^3^ 51.25 ± 15.85 ^3^	RCT, double-blind	Not mentioned	Not mentioned
Lin 2024 [18]	Taiwan	ESRD under HD	Omega-3: 14/16 ^2^ Placebo: 20/10 ^2^	66.63 ± 11.67 ^2^ 67.57 ± 11.57 ^2^	RCT	Not mentioned	Not mentioned

CAPD, continuous ambulatory peritoneal dialysis; ESRD, End-stage renal disease; HD, hemodialysis; RCT, randomized controlled trial; USA, United States of America. ^1^ presented as mean ± standard deviation. ^2^ Allocated participants. ^3^ Per-protocol participants. ^4^ Subjects from the safflower oil and olive oil groups were merged into one group and treated as the experimental control group for statistical analysis. ^5^ The numbers for the male-to-female ratio and total number of participants in the omega-3 fatty acid group and the control group appear to have been switched in the original article.

**Table 2:** Summary of the omega-3 fatty acids interventions delivered in the study treatment arms of the studies. The table describes the seven trials enrolled for meta-analysis. Key parameters of these trials are summarized in the table. The intervention duration is the same as the period for meta-analysis.

First Author and Year	Dialysis Vintage ^1^ (Month/Year)	Intervention Duration	Omega-3 Fatty Acid Product/Manufacturer	Daily Omega-3 FA/DHA/EPA Dose (Per-Protocol N)	Control (Per-Protocol N)	Pruritus Outcome Measurement (Score Range)
Peck 1996 [19]	Not mentioned	8 weeks	National Oceanic and Atmospheric Administration, Charleston Laboratory	6 gm/n.a./n.a. (8)	olive oil (9) safflower oil (8)	Developed by Duo (0–10) [54] ^2^
Begum 2004 [20]	Omega-3 58.0 ± 40.2 m Placebo 70.5 ± 55.6 m	16 weeks	National Oceanic and Atmospheric Administration, Charleston Laboratory	6 gm/n.a./n.a. (12)	α-tocopherol and γ-tocopherol as a placebo (10)	Developed by Duo (0–40) [54] ^3^
Ghanei 2012 [22]	Omega-3 45.7 ± 24.4 m Placebo 61.0 ± 58.5 m	20 days	Zahravi Pharmaceutical Company, Tabriz, Iran	3 gm/0.36 gm/0.54gm (11)	Placebo capsule (11)	Developed by Duo (0–40) [54] ^3^
Lahiji 2018 [21]	Omega-3 38.0 ± 22.8 m Placebo 37.6 ± 23.3 m	4 weeks	Zahravi Pharmaceutical Company, Tabriz	3 gm/0.36 gm/0.54 gm (20)	Placebo capsule (20)	Pruritus VAS score (1–10)
Shayanpour 2019 [23]	more than three months	3 weeks	Not mentioned	2 gm/n.a./n.a. (32)	Placebo capsule (32)	5D pruritus questionnaire scale (5–25) [55]
Forouhari 2022 [17]	Omega-3 37.8 ± 31.7 m Placebo 29.5 ± 13.8 m	4 weeks	Zahravi Pharmaceutical Company, Tabriz, Iran	3 gm/0.36 gm/0.54 gm (17)	Placebo capsule (16)	Pruritus VAS score (1–10)
Lin 2024 [18]	Omega-3 59.6 ± 38.4 m Placebo 76.4 ± 36.8 m	3 months	Chen Hua Biotech Co., LTD, Taoyuan, Taiwans	2 gm/0 gm/0.9 gm (30)	Soybean capsule as placebo (30)	5D pruritus questionnaire scale (5–25) (53)

FA, fatty acid; n.a., not available; VAS, Visual Analog Scale. ^1^ presented as mean ± standard deviation. ^2^ The severity score was adapted for outcome measurement. ^3^ The overall score was adapted for outcome measurement.

**Table 3:** Detailed quality appraisal of included studies using the Cochrane risk of bias tool for randomized trials (RoB 2). The table summarizes the level of risk from different perspectives, including randomization process, intervention adherence, missing data outcome, outcome measurement, selecting reporting, and overall risk of bias. Superscripted numbers indicate the details and reasons of the assessment.

First Author	Year	Randomization Process	Intervention Adherence	Missing Outcome Data	Outcome Measurement	Selective Reporting	Overall Risk of Bias
Peck [19]	1996	S ^1^	L	L	L	L	S
Begum [20]	2004	S ^1^	L	L	L	L	S
Ghanei [22]	2012	S ^1^	L	L	L	L	S
Lahiji [21]	2018	H ^2^	L	L	L	L	S
Shayanpour [23]	2019	L	L	L	L	L	L
Forouhari [17]	2022	S ^1^	L	L	L	L	S
Lin [18]	2024	S ^1^	S	L	L	L	S

H, high risk of bias; L, low risk of bias; S, some concerns of risk of bias. ^1^ The studies did not provide allocation concealment details. ^2^ The study revealed an imbalance in baseline visual analog scale pruritus score between the study and controls.

## Data Availability

The original contributions presented in this study are included in the article/Supplementary Materials. Further inquiries can be directed to the corresponding author.

## Supplementary Materials

The following supporting information can be downloaded at: https://www.mdpi.com/article/10.3390/ph19010181/s1. Figure S1: Adverse events. Figure S2: Funnel plot. Table S1: Studied excluded from the meta-analysis and reasons of exclusion. Table S2: Keywords and field tags for literature searching for article identification. Table S3: Variables and information on the trials of using omega-3 fatty acids for uremic pruritus. PRISMA 2020 checklist [43].
