# Supplementary material for: Effectiveness of Eicosapentaenoic and Docosahexaenoic Acid Supplementation for Reducing Uremic Pruritus: A Meta-Analysis of Randomized Controlled Trials
Source: Pharmaceuticals (Basel). 2026 Jan 20;19(1):181. doi: 10.3390/ph19010181 (PMC12844951; doi:10.3390/ph19010181)
Supplement: Supplementary file 1 [file pharmaceuticals-19-00181-s001.zip › pharmaceuticals-4005219-Supplementary Materials/Table S1 - excluded articles.pdf]

Table S1. Studied excluded from the meta-analysis and reasons of exclusion

First author & year	Title	Reasons of exclusion
Heydarbaki 2020 [51]	The effects of omega-3 on the sleep quality of patients with uremic pruritus undergoing hemodialysis: a randomized crossover study.	Comparing the effect between omega-3 plus cetirizine and cetirizine. Lack of a placebo.
Heydarbaki 2021 [53]	An Investigation into the Effects of Omega-3 on Uremic Pruritus in Hemodialysis Patients: A Randomized Crossover Clinical Trial	Comparing the effect between omega-3 plus cetirizine and cetirizine. Lack of a placebo.
Lin 2022 [38]	Omega-3 Fatty Acids Improve Chronic Kidney Disease-Associated Pruritus and Inflammation	Single-arm trial
Rafieipoor 2024 [52]	Effectiveness of omega-3 fatty acid supplementation for pruritus in patients undergoing hemodialysis.	Lack of pruritus score as primary outcome measurement for this meta-analysis
Teama 2025 [49]	Omega-3 fatty acids versus gabapentin in uremic pruritus in hemodialysis patients: randomized controlled, crossover clinical trial.	Comparing the effect between omega-3 and gabapentin. Lack of a placebo.
