# Supplementary material for: Effectiveness of Eicosapentaenoic and Docosahexaenoic Acid Supplementation for Reducing Uremic Pruritus: A Meta-Analysis of Randomized Controlled Trials
Source: Pharmaceuticals (Basel). 2026 Jan 20;19(1):181. doi: 10.3390/ph19010181 (PMC12844951; doi:10.3390/ph19010181)
Supplement: Supplementary file 1 [file pharmaceuticals-19-00181-s001.zip › pharmaceuticals-4005219-Supplementary Materials/Table S2 - search keywords and field tags.pdf]

Table S2 Keywords and field tags for literature searching for article identification

Database	Search keywords and field tags
Pubmed	("Fish Oils"[Mesh] OR fish oil*[tiab] OR "Omega-3 Fatty Acids"[Mesh] OR omega-3 fatty acid*[tiab] OR omega 3[tiab] OR n-3 fatty acid*[tiab] OR fish oil*[tiab] OR "Eicosapentaenoic Acid"[Mesh] OR eicosapentaenoic acid*[tiab] OR EPA[tiab] OR "Docosahexaenoic Acids"[Mesh] OR docosahexaenoic acid*[tiab] OR DHA[tiab]) AND ("Pruritus"[Mesh] OR pruritus[tiab] OR itch*[tiab] OR uremic pruritus[tiab] OR uraemic pruritus[tiab] OR "Chronic Kidney Failure"[Mesh] OR "Renal Insufficiency, Chronic"[Mesh] OR chronic kidney disease[tiab] OR CKD[tiab] OR end-stage renal disease[tiab] OR ESRD[tiab] OR uremia[tiab] OR uraemia[tiab])
EMBASE	("Pruritus"[Mesh] OR pruritus[tiab] OR itch*[tiab] OR uremic pruritus[tiab] OR uraemic pruritus[tiab] OR "Chronic Kidney Failure"[Mesh] OR "Renal Insufficiency, Chronic"[Mesh] OR chronic kidney disease[tiab] OR CKD[tiab] OR end-stage renal disease[tiab] OR ESRD[tiab] OR uremia[tiab] OR uraemia[tiab]) EMBASE ("Fish Oils":ti,ab OR "Omega-3 Fatty Acids":ti,ab OR "n-3 fatty acid*":ti,ab OR "Eicosapentaenoic Acid":ti,ab OR "EPA":ti,ab OR "Docosahexaenoic Acid":ti,ab OR "DHA":ti,ab) AND ("Pruritus":ti,ab OR "itch":ti,ab OR "uremic pruritus":ti,ab OR "Chronic Kidney Failure":ti,ab OR "chronic kidney disease":ti,ab OR "CKD":ti,ab OR "end-stage renal disease":ti,ab OR "ESRD":ti,ab OR "uremia":ti,ab OR "uraemia":ti,ab)
Cochrane central	("Fish Oils":ti,ab,kw OR "Omega-3 Fatty Acids":ti,ab,kw OR "n-3 fatty acid*":ti,ab,kw OR "Eicosapentaenoic Acid":ti,ab,kw OR "EPA":ti,ab,kw OR "Docosahexaenoic Acid":ti,ab,kw OR "DHA":ti,ab,kw) AND ("Pruritus":ti,ab,kw OR "itch":ti,ab,kw OR "uremic pruritus":ti,ab,kw OR "Chronic Kidney Failure":ti,ab,kw OR "chronic kidney disease":ti,ab,kw OR "CKD":ti,ab,kw OR "end-stage renal disease":ti,ab,kw OR "ESRD":ti,ab,kw OR "uremia":ti,ab,kw OR "uraemia":ti,ab,kw)
Clinicaltrial.gov	("Fish Oil" OR "Omega-3 Fatty Acids" OR "n-3 fatty acids" OR "Eicosapentaenoic Acid" OR "EPA" OR "Docosahexaenoic Acid" OR "DHA") AND ("Pruritus" OR "Itch" OR "Uremic Pruritus" OR "Chronic Kidney Disease" OR "CKD" OR "Chronic Kidney Failure" OR "End-Stage Renal Disease" OR "ESRD" OR "Uremia" OR "Uraemia")
