# Supplementary material for: Effectiveness of Eicosapentaenoic and Docosahexaenoic Acid Supplementation for Reducing Uremic Pruritus: A Meta-Analysis of Randomized Controlled Trials
Source: Pharmaceuticals (Basel). 2026 Jan 20;19(1):181. doi: 10.3390/ph19010181 (PMC12844951; doi:10.3390/ph19010181)
Supplement: Supplementary file 1 [file pharmaceuticals-19-00181-s001.zip › pharmaceuticals-4005219-Supplementary Materials/Table S3 - datas and trials variables.pdf]

| First author & year | Country | Population | Participants (female/male) | Age [1] | Study design | Allocation concealment | Randomization | Funding/grants/support |
|---|---|---|---|---|---|---|---|---|
| Peck 1996 | USA | ESRD under HD | Omega-3: 3/5 [2]<br>Olive oil: 5/4 [2,4]<br>Safflower oil: 4/4 [2,4] | 54.8±16.2<br>45.6±17.4<br>49.5±17.2 | RCT, double-blind | Not mentioned | Not mentioned | Northwest Kidney Foundation, Seattle. |
| Begum 2004 | USA | ESRD under HD | Omega-3: 3/7 [2,5]<br>Placebo: 6/6 [2,5] | 61.2±19.42<br>49.25±18.12 | RCT, double-blind | Not mentioned | Not mentioned | National Kidney Foundation, Inc. |
| Ghanei 2012 | Iran | ESRD under HD | Omega-3: 3/8 [2]<br>Placebo: 5/6 [2] | 59.90±14.82<br>53.09±13.08 | RCT, double-blind | Not mentioned | Not mentioned | Dr. Bagheri and Zahravi Pharmaceutical Company supplied omega-3 FAs and the placebo. |
| Lahiji 2018 | Iran | ESRD under CAPD | Omega-3: 11/9 [2]<br>Placebo: 10/10 [2] | 62.1±11.6<br>61.9±10.8 | RCT, double-blind | Not mentioned | Not mentioned | Nil |
| Shayanpour 2019 | Iran | ESRD under HD | Omega-3: 5/27 [2]<br>Placebo: 9/23 [2] | 51.91± 6.58<br>56.25±8.86 | RCT, double-blind | Blocks of six for allocation concealment | Blocks of six for allocation concealment | Ahvaz Jundishapur University of Medical Sciences. |
| Forouhari 2022 | Iran | ESRD under HD | Omega-3: 5/12 [3]<br>Placebo: 5/11 [3] | 59.00±13.56 [3]<br>51.25±15.85 [3] | RCT, double-blind | Not mentioned | Not mentioned | Not mentioned |
| Lin 2024 | Taiwan | ESRD under HD | Omega-3: 14/16 [2]<br>Placebo: 20/10 [2] | 66.63±11.67 [2]<br>67.57±11.57 [2] | RCT | Not mentioned | Not mentioned | Grants from HungKuang University and Kuang Tien General Hospital (HK-KTOH-110–04) |

**Table S3 Variables and information on the trials of using omega-3 fatty acids for uremic pruritus.**

The table describes the seven trials included in the meta-analysis. Key information on the study designs is summarized in the table. CAPD: continuous ambulatory peritoneal dialysis; ESRD: End-stage renal disease; HD: hemodialysis; RCT, randomized controlled trial; USA, United States of America

[1] presented as mean ± standard deviation.

[2] Allocated participants

[3] Per-protocol participants

[4] Subjects from the safflower oil and olive oil groups were merged into one group and treated as the experimental control group for statistical analysis.

[5] The numbers for the male-to-female ratio and total number of participants in the fish oil group and the control group appear to have been switched in the original article.
