# Supplementary figures and images for: Effectiveness of Eicosapentaenoic and Docosahexaenoic Acid Supplementation for Reducing Uremic Pruritus: A Meta-Analysis of Randomized Controlled Trials
Source: Pharmaceuticals (Basel). 2026 Jan 20;19(1):181. doi: 10.3390/ph19010181 (PMC12844951; doi:10.3390/ph19010181)

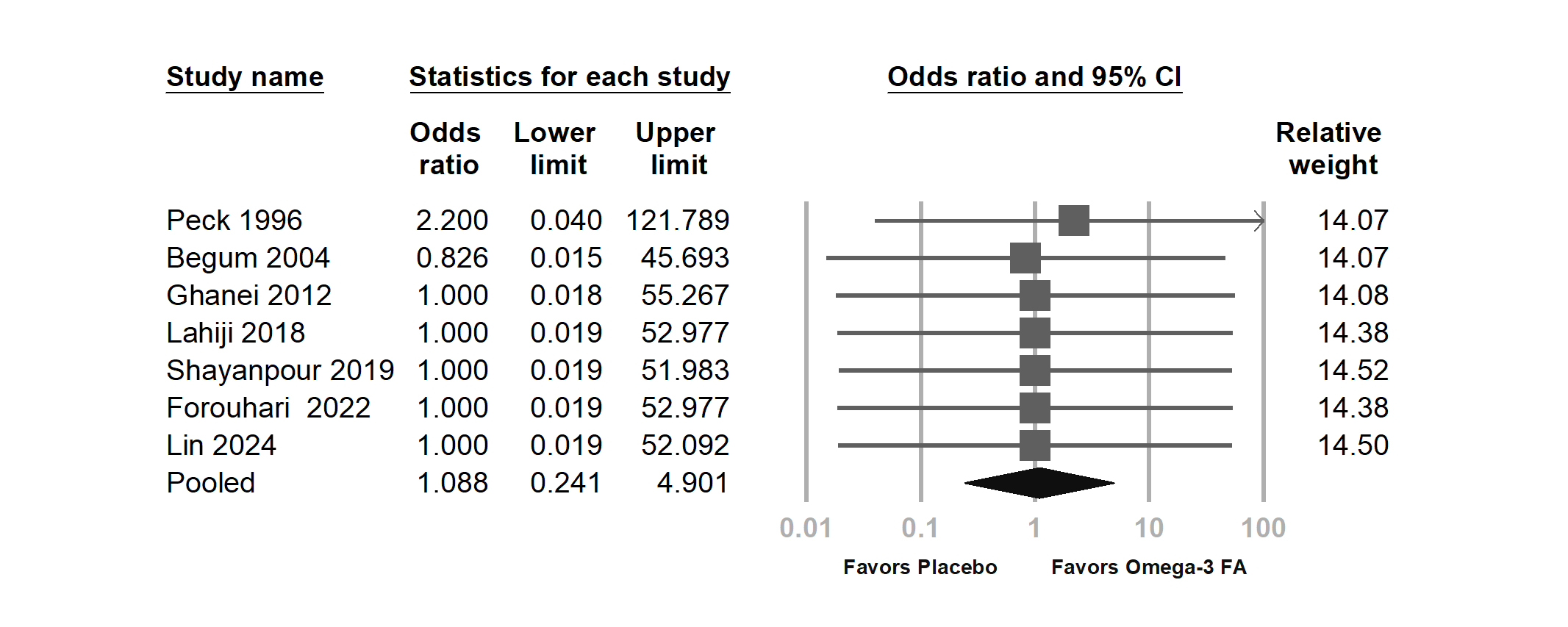

Supplement: Supplementary file 1 [file pharmaceuticals-19-00181-s001.zip › pharmaceuticals-4005219-Supplementary Materials/Figure S1 adverse events.tif]

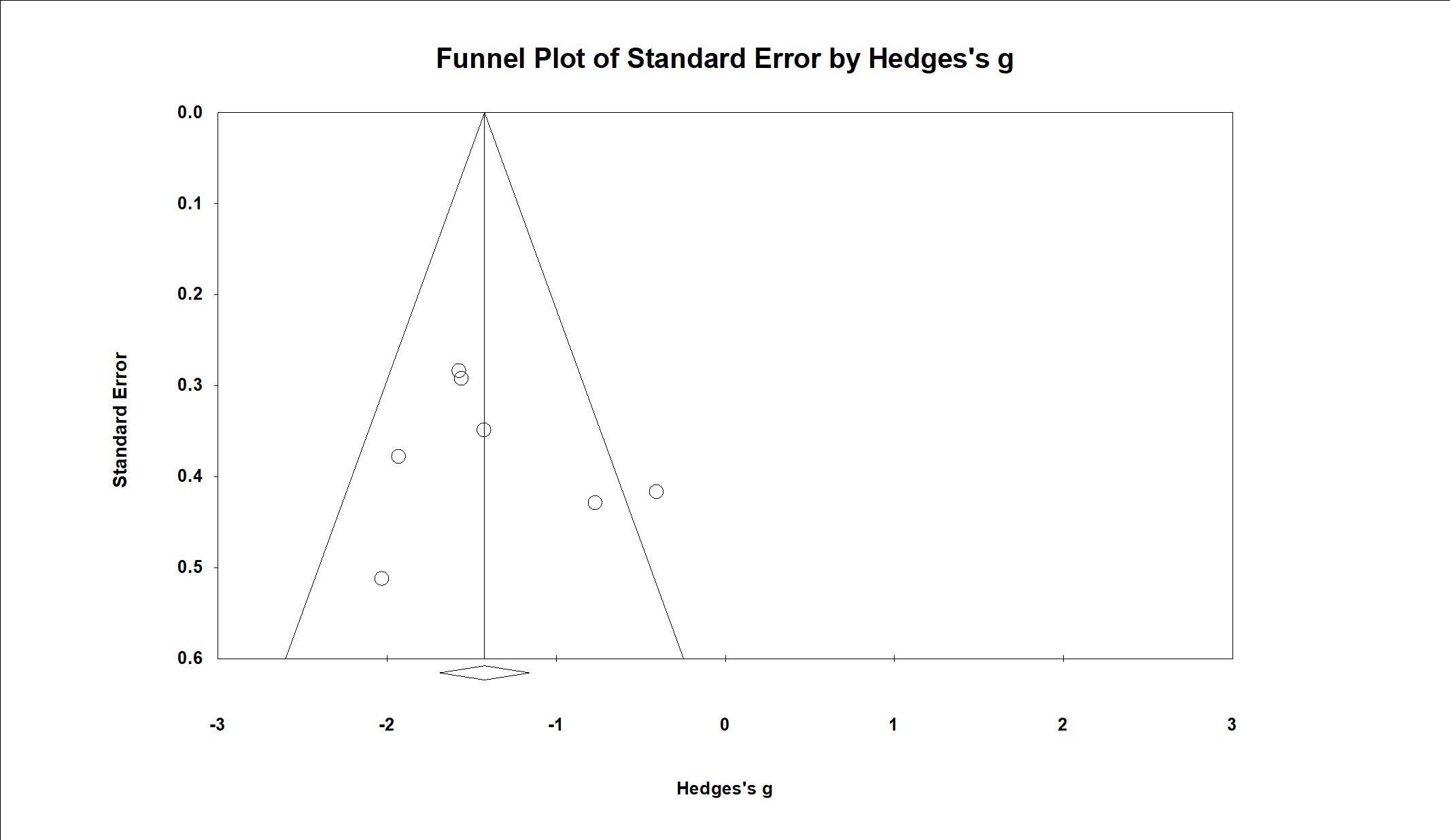

Supplement: Supplementary file 1 [file pharmaceuticals-19-00181-s001.zip › pharmaceuticals-4005219-Supplementary Materials/Figure S2 funnel plot.tif]
